# Development and laboratory evaluation of a competitive ELISA for serodiagnosis of Nipah and Hendra virus infection using recombinant Nipah glycoproteins and a monoclonal antibody

**DOI:** 10.3389/fvets.2023.1120367

**Published:** 2023-02-01

**Authors:** Wenjun Zhu, Bradley Pickering, Greg Smith, Mathieu Pinette, Thang Truong, Shawn Babiuk, Darwyn Kobasa, Logan Banadyga, Ming Yang

**Affiliations:** ^1^National Centre for Foreign Animal Disease, Canadian Food Inspection Agency, Winnipeg, MB, Canada; ^2^Department of Medical Microbiology and Infectious Diseases, University of Manitoba, Winnipeg, MB, Canada; ^3^Department of Veterinary Microbiology and Preventative Medicine, College of Veterinary Medicine, Iowa State University, Ames, IA, United States; ^4^National Microbiology Laboratory, Public Health Agency of Canada, Winnipeg, MB, Canada; ^5^Department of Immunology, University of Manitoba, Winnipeg, MB, Canada

**Keywords:** Nipah virus, Hendra virus, Henipavirus, recombinant Nipah virus glycoprotein, monoclonal antibody, competitive ELISA, serodiagnosis

## Abstract

**Introduction:**

Nipah virus (NiV) and Hendra virus (HeV), of the genus *Henipavirus*, family *Paramyxoviridae*, are classified as Risk Group 4 (RG4) pathogens that cause respiratory disease in pigs and acute/febrile encephalitis in humans with high mortality.

**Methods:**

A competitive enzyme–linked immunosorbent assay (cELISA) using a monoclonal antibody (mAb) and recombinant NiV glycoprotein (G) was developed and laboratory evaluated using sera from experimental pigs, mini pigs and nonhuman primates. The test depends on competition between specific antibodies in positive sera and a virus–specific mAb for binding to NiV–G.

**Results:**

Based on 1,199 negative and 71 NiV positive serum test results, the cutoff value was determined as 35% inhibition. The diagnostic sensitivity and specificity of the NiV cELISA was 98.58 and 99.92%, respectively. When testing sera from animals experimentally infected with NiV Malaysia, the cELISA detected antibodies from 14 days post–infection (dpi) and remained positive until the end of the experiment (28 dpi). Comparisons using the Kappa coefficient showed strong agreement (100%) between the cELISA and a plaque reduction neutralization test (PRNT).

**Discussion:**

Because our cELISA is simpler, faster, and gives comparable or better results than PRNT, it would be an adequate screening test for suspect NiV and HeV cases, and it would also be useful for epidemiological surveillance of Henipavirus infections in different animal species without changing reagents.

## Introduction

Nipah virus (NiV) and Hendra virus (HeV) are enveloped, negative–sense RNA viruses that belong to the genus *Henipavirus* in the virus family *Paramyxoviridae*. These zoonotic viruses cause fatal diseases in animals and humans (1–3). NiV was first observed in Malaysia and Singaporean pigs in 1998–99, with 105 confirmed human deaths. Since then, NiV infections have been identified in both animals and humans in South and Southeast Asia (1, 4, 5). HeV was first identified during a disease outbreak in 1994 in Australia, where several horses and their trainer died from a pulmonary disease with hemorrhagic manifestations (6). NiV and HeV have been identified as high priority pathogens by WHO due to their broad geographical distribution, their potential to cause severe disease and the lack of countermeasures. The host reservoirs of Henipaviruses include fruit bats, domestic pigs, cats, dogs and horses (1). Transmission is supposed to occur from bats *via* saliva, urine, and excreta to humans, with pigs (NiV) or horses (HeV and NiV) as intermediate hosts. Spillover transmission from bats to intermediate hosts or humans is due to consumption of contaminated fruits or exposure to contaminated secretions (1, 7, 8).

Typically, for the diagnosis of infectious diseases, serology is used to determine prior viral exposure and immunity to specific pathogens. Therefore, it is commonly used for viral infection confirmation and surveillance in the community and in livestock. Reliable detection of specific antibodies is also important to better understand the ecology of fruit bat hosts and the risk of transmission to other intermediate hosts. Therefore, it can facilitate the implementation of methods for the prevention and management of human and animal outbreaks (9).

Currently, the plaque reduction neutralization test (PRNT) is considered the “gold standard” for detecting and measuring NiV– and HeV– specific antibodies that can neutralize viruses. However, the neutralization test requires live viruses and a high–security Biosafety Level 4 (BSL−4) facility. To overcome these disadvantages, a serum neutralization test was reported for measuring NiV neutralizing antibodies under BSL2 conditions using a recombinant vesicular stomatitis virus (VSV) expressing NiV fusion protein (F) and glycoprotein (G) (10), as well as NiV and HeV F and G proteins in Moloney murine leukemia virus (11). However, neutralization tests are still labor–intensive and time consuming, making large–scale serology testing difficult. ELISA is a simple and rapid procedure that often provides more reproducible results. Several indirect ELISAs for detecting NiV and HeV antibodies have been reported previously (3, 12–14). The disadvantage of indirect ELISA is that species–specific secondary antibodies are required for different animal species and humans. Because NiV and HeV are zoonotic viruses, they infect and cause severe illness and death in animals and humans. Thus for diagnoses and surveillances, changing secondary antibodies for different species is inconvenient, and test conditions may need to be changed and re–titrated. To overcome the limitations mentioned for indirect ELISA, competitive ELISAs (cELISAs) are commonly used for antibody detection due to their sensitivity, simplicity, ease to scale up to accommodate the screening of large numbers of serum samples, and suitability to detect antibodies from different species without needing species–specific secondary antibodies. Although a solid–phase blocking ELISA for the detection of NiV antibodies has been reported, this assay relied on NiV–infected cells as antigen (15).

The present study aimed to develop a simple and rapid serodiagnostic assay for pan–henipavirus (NiV, HeV) antibody detection. A cELISA was developed using the Baculovirus expressed recombinant NiV–G antigen, and a NiV–G specific monoclonal antibody (mAb). The NiV cELISA was evaluated using negative and henipavirus–positive serum samples from experimentally inoculated pigs, mini pigs and non-human primates (NHPs). Importantly, this cELISA can detect either NiV and/or HeV antibodies from different animal species, demonstrating the assay is suitable for use in diagnosing henipavirus infection, monitoring the effectiveness of vaccinations, and performing epidemiological surveillance.

## Materials and methods

### Ethics statement

Animal experimental design, including housing conditions, sampling regimen, and humane endpoints, was approved by the Animal Care Committee of the Canadian Science Centre for Human and Animal Health in #C−02–006, #C−04–005, and #C−08–008. All procedures were performed in strict accordance with the Canadian Council on Animal Care guidelines. Group housing was carried out in the CL 4 animal cubicle, and animals were provided with commercial toys for enrichment and access to food and water *ad libitum*. The animal studies reported in this manuscript complied with the Animal Research: Reporting of *in vivo* Experiments (ARRIVE) guidelines.

### Recombinant his–tagged Nipah virus soluble glycoprotein expression

Full–length NiV glycoprotein (G) expression was previously described (16). Briefly, the full–length glycoprotein of NiV Bangladesh strain (B) lacking the cell membrane and transmembrane domains (GenBank: AY988601.1) was synthesized with codon optimization, and cloned into pAB–bee™ –FH vector containing a polyhedron promoter (AB Vector, LLC, San Diego, CA). The purified pAB–bee™ –FH vector containing the NiV–G gene was co–transfected with Baculovirus vector DNA, ProFold™–ER1 (AB Vector, LLC, San Diego, CA) into Sf9 insect cells (Expression System) to generate the recombinant Baculovirus NiV–G gene. Individual clones expressing the NiV–G gene, were plaque purified using SF9 cells. These clones were then subcultured and therefore expressed in Trichoplusia ni (Tni) cells (Expression Systems). The infected cells were harvested 72 hours post–infection; recombinant NiV G was purified, and expression was confirmed by Western blot analysis.

### Generation of monoclonal antibodies to Nipah virus

Hybridoma cells were generated as previously described (17). Briefly, female Balb/c mice were inoculated subcutaneously with purified NiV–M (20–30 μg/mouse) in an equal volume of complete Freund's adjuvant and incomplete Freund's adjuvant (Difco, BD, Oakville, ON, CA). Three identical inoculations were performed, followed by a final boost 3 days before fusion. Spleen cells from immunized mice were fused with myeloma cells (P3X63 Ag8.653, ATCC, Rockville, MD, USA). Hybridoma supernatants were screened using NiV–M indirect ELISA. The positive clones were subcloned and the mAbs were isotyped using a mouse monoclonal antibody isotyping kit (Roche, Indianapolis, IN, USA).

### Virus preparation

Vero E6 (African green monkey kidney) cells were cultured in Dulbecco's minimal essential medium (DMEM, Sigma–Aldrich, SL, USA) supplemented with 10% fetal bovine serum (FBS) and 2 mM L–glutamine (Life Technologies). The NiV Malaysia strain (NiV–M) and Hendra virus, derived from clinical isolates, were generously provided by the US Center for Disease Control (CDC) and were passaged a total of 3 passages in Vero E6 prior to being used in experiments. A third passage stock of the NiV–B was generated from the reverse genetics–derived virus as previously described (18).

### Non-human primate (NHP) experiment and serum collection

Twelve cynomolgus macaques (six male and six female), weight range of 3.75 to 5 kg, were divided into 3 groups of 4 animals (2 male and 2 female) and one group was infected with each of the three NiV–B, NiV–M and HeV. All animals were infected by intramuscular injection in the thigh with 10^5^ plaque–forming units in 0.5 ml of neat Minimal Essential Medium (MEM). Blood was collected, once before infection and after infection 14 and 28 days post–infection (dpi).

### Other serum samples

The Nipah virus (–B, –M), Hendra virus, and Ebola virus–positive samples from pigs and miniature (mini) pigs used in this study were from previously reported experimental studies at the National Center for Foreign Animal Disease and Public Health Agency of Canada (19–21). The NiV and HeV positive samples were confirmed positive using either PRNT or indirect ELISAs. Negative serum samples were collected from naïve controls in experimental animal studies and farms in Manitoba, Canada.

### Plaque reduction neutralization test

The method for PRNT using live NiV was described previously (19), and all procedures with the live virus were performed under BSL−4 conditions. Briefly, mixtures of virus and dilutions of pig sera were incubated at 37°C for 1 h, then added to Vero cell monolayers. After incubation at 37°C for 1 h, a 1.75% Carboxy–methyl cellulose (Sigma–Aldrich, SL, USA) overlay was added, and the plates were placed in a CO_2_ incubator for 72 h. Then the cells were fixed with 4% formaldehyde for 24 h and stained with 0.5% crystal violet plus 80% methanol in phosphate–buffered saline (PBS).

### Indirect ELISAs for the detection of antibodies against NiV–G protein

The indirect ELISA was performed as previously described (20). Briefly, Nunc 96–Well Microplates were coated with soluble NiV–G (20 ng/well) in 0.06 M carbonate/bicarbonate buffer (pH 9.6) overnight at 4°C. Plates were washed five times with 0.05% Tween 20 in PBS (PBS–T) and blocked with a blocking buffer (3% BSA/10% horse serum in PBS–T). Serum samples (1:100) were added, and then the enzyme horseradish peroxidase (HRP)–goat anti–Swine or anti–NHP IgG antibody (SeraCare Life Sciences, MA, USA) was added. Reactions were developed using 2,20–azino–bis[3—thylbenzothiazoline−6–sulphonic acid] (Roche Diagnostics, IN, USA) and read at 405 nm on a BioTek Epoch Microplate Spectrophotometer. Each incubation step was 1 h at 37°C and followed by washing five times with the washing buffer.

### Competitive ELISA

Nunc 96–Well non–surface treated Microplates (Thermo Fisher Scientific, MA, USA) were coated with 100 μl (12 ng/well) of the recombinant NiV–G in 0.06 M carbonate/bicarbonate buffer (pH 9.6) overnight at 4°C. After washing 5 times with PBS–T, plates were blocked with 5% commercial porcine serum (Life Technologies, New Zealand) in Casein blocking buffer (Sigma–Aldrich, SL, USA). A commercial normal pig serum was used as a negative control, and one serum collected at 28 dpi from NiV–B inoculated pig (#7) was used as a positive control in the cELISA. Negative and positive serum controls are included in each ELISA plate to calculate percent inhibition (PI) results for test samples. Thus after blocking, test serum samples, positive, and negative serum controls (50 μl/well, 1:10 in PBS–T) were added in duplicate, then an equal volume of the hybridoma culture supernatant (F20NiV−65, 1:500 in Casein blocking buffer) were added at the same time. Following 1 h incubation and washing, the HRP–conjugated anti–mouse IgG (1:500, Jackson ImmunoResearch Laboratories, West Grove, PA, USA) was added. Then 3,3′,5,5′–Tetramethylbenzidine (TMB, Pierce Biotechnology Inc. IL, USA) was added. The reaction was stopped using 2N Sulfuric acid and the OD was determined at 450 nm using a BioTek Epoch Microplate Spectrophotometer reader. Results of PI were calculated based on the following formula:


$$\begin{array}{l} \text{PI }\left(\%\right)=\left[\frac{\left(\text{OD of negative control well }-\text{ OD of test sample well}\right)}{\left(\text{OD of negative control well }-\text{ OD of positive control well}\right)\;}\right]\\ \text{ X }100 \end{array}$$

### Statistical analysis

Calculations of mean values, standard deviations and coefficients of linear regression were done as standard descriptive procedures. The GraphPad Prism 9 software was used to calculate the receiver operating characteristic curve (ROC) curve and determine the cutoff value. In ROC calculation, true positive sera were defined as those tested using NiV–G indirect IgG ELISA from experimentally inoculated animals. Cohen's kappa coefficient was calculated to evaluate interrater agreement between cELISA and PRNT using Excel.

## Results

### Antibody reactivity to recombinant Nipah virus glycoprotein

**Figure 1:**
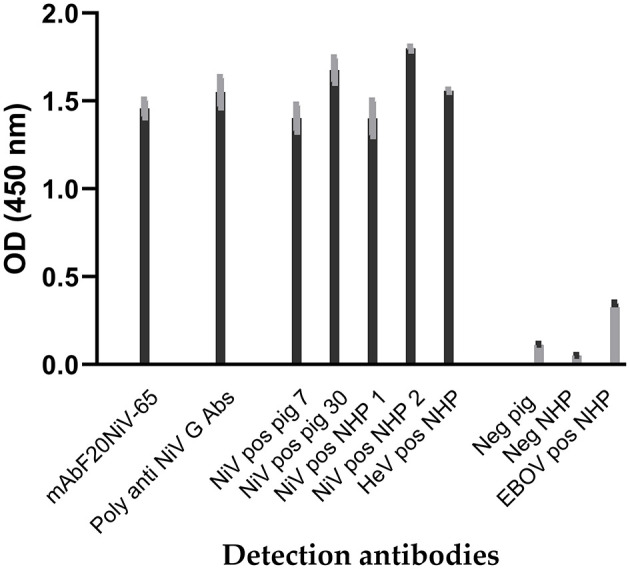
Reactivity of Nipah virus and Hendra virus–positive sera to recombinant Nipah virus glycoprotein in indirect ELISA. Recombinant Nipah virus glycoprotein (NiV–G) was coated onto ELISA plates. Then NiV, Hendra virus (HeV), or Ebola virus (EBOV) positive sera from experimentally inoculated pigs and non-human primate (NHP) were added. Negative sera, a polyclonal anti–NiV–G antibody, and a NiV–specific mAb (F20NiV−65) were used as controls. The antibody binding was detected with HRP–conjugated anti–swine, anti–NHP, or anti–mouse antibodies. The data shown are the mean of duplicates with error bars.

Recombinant NiV–G was produced in insect cells infected with recombinant Baculovirus expressing the NiV–G gene and purified. Antibody responses to recombinant NiV–G were examined using polyclonal and monoclonal antibodies to confirm that recombinant NiV–G contained immunodominant epitopes. Recombinant NiV–G was tested using a porcine NiV–positive serum. The result demonstrated a molecular weight of approximately 70 kDa using Western blot analysis (16). In indirect ELISA, both NiV–specific monoclonal and polyclonal antibodies, as well as NiV– and HeV–positive sera from pigs and NHPs showed binding to the recombinant NiV–G (Figure 1). Negative sera and Ebola virus glycoprotein–positive serum demonstrated negative reactivity to NiV–G. NiV and HeV polyclonal sera responded similarly to NiV–G. The results indicate that recombinant NiV–G contained immunodominant epitopes that are conserved for both NiV and HeV. Thus, the NiV–G was selected and used as the antigen in cELISA.

### Development of cELISA for NiV and HeV antibody detection

**Figure 2:**
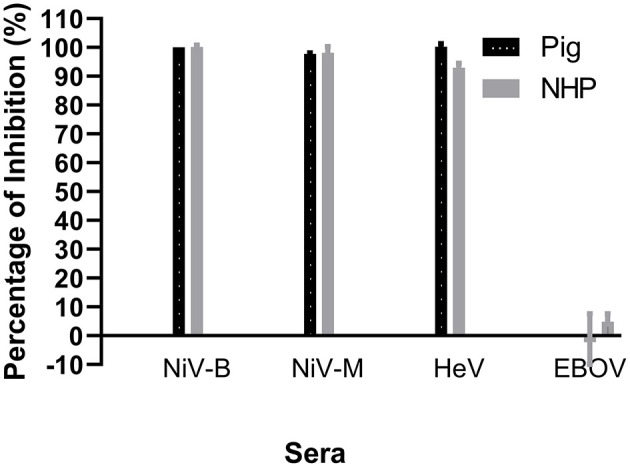
Evaluation of NiV cELISA for the detection of antibodies against Nipah and Hendra viruses. Recombinant NiV–G was coated onto ELISA plates. Then NiV (Bangladesh, Malasia), HeV, or Ebola virus (EBOV) positive sera from experimentally inoculated animals was added, followed by NiV–specific mAb F20NiV−65. The HRP–conjugated anti–mouse IgG antibody was added, followed by the TMB substrate. The percentage inhibition was calculated based on the negative serum. The data shown are the mean of duplicates with error bars.

Since the NiV–specific mAb F20NiV−65 was determined to react with recombinant NiV–G, its ability to compete with NiV–positive sera for binding to NiV–G was tested in a cELISA. The results showed that this mAb had a strong inhibitory effect on the binding of a pig NiV–positive serum to NiV–G (data not shown). Therefore, the mAb F20NiV−65 was chosen as a competitor in the cELISA. Optimal coating antigen concentration, antibody dilution, and buffer selection were determined by checkerboard titration and comparative experiments to ensure maximum differences between positive and negative control sera. The optimal dilution of mAb was 1:500, while the preferred serum dilution was 1:10. A cELISA was performed to determine whether the NiV cELISA could similarly detect HeV, NiV (B and M) and HeV antibodies in positive pig and NHP sera. The results showed high percentage inhibition with NiV and HeV positive sera (Figure 2), which confirmed that the NiV cELISA is suitable for detecting NiV and HeV antibodies in different animal species without changing reagents.

### Determination of specificity and sensitivity

A total of 1199 sera (pig *n* = 1171, NHP *n* = 28) collected from Canadian farms (pigs) or pre–bleeds of animal experiments (NHPs) were used as true negative samples, and 71 NiV or HeV true positive sera from experimentally infected animals (19, 20) were used to determine the diagnostic sensitivity, specificity and threshold cutoff of NiV cELISA.

**Figure 3:**
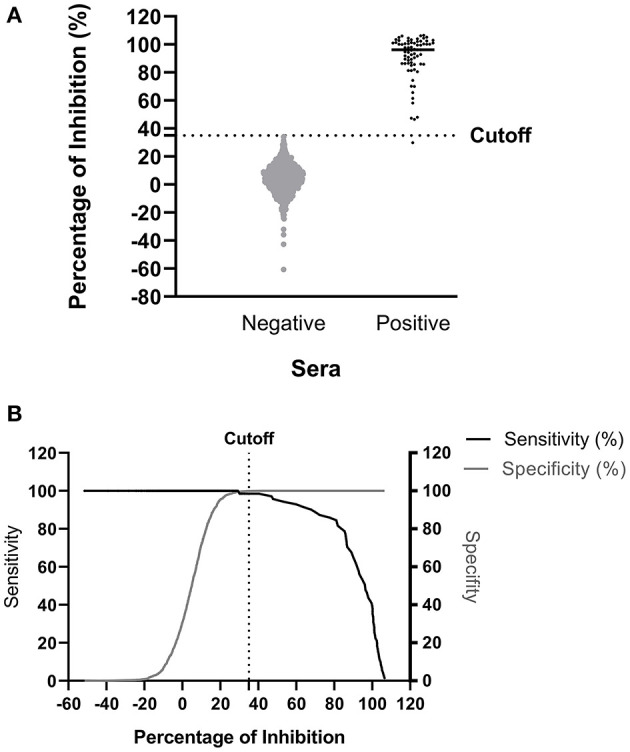
Determination of diagnostic sensitivity and specificity of the NiV cELISA using receiver operating characteristic curve. A total of 71 NiV or HeV positive sera and 1199 negative sera were tested using the NiV cELISA, and data were analyzed using receiver operating characteristic curve (ROC) **(A)** Dot plot of the NiV cELISA results for both negative and positive sera. The horizontal dotted line represents the cutoff value (35.45%) that gave the best diagnostic sensitivity and specificity **(B)** Graph of diagnostic sensitivity and specificity using ROC curve. The dotted line represents the cutoff value (35%) for percent inhibition.

The ROC analysis shows the optimal cutoff values and corresponding sensitivity and specificity of the NiV cELISA in Figures 3A, B. The cutoff value for ROC determination was 35%, which was nearly identical to the cutoff of 35.45% that was calculated based on the mean PI of negative sera plus three standard deviations. The results of negative sera in pigs and non-human primates (NHPs) using NiV cELISA are summarized in Table 1. Therefore, samples with a PI value equal to or >35% were considered positive. The diagnostic sensitivity of NiV cELISA was 98.59% and the diagnostic specificity was 99.92%. One serum sample (14 dpi) from an NHP experimentally inoculated with HeV was positive using NiV indirect ELISA, but was detected as negative result using the NiV cELISA (PI = 29.9%). This sample was not tested using PRNT (Table 2).

**Table 1:** Summary of negative sera in pig and non-human primate (NHP) test results using NiV cELISA.

**Animal species**	**Total sample number**	Mean PI^*^(%)	**Standard deviation**	**Cut– off value (PI%)**
Pig	1,171	4.63	10.27	35.46
NHP	28	−5.90	8.06	18.27
Pig + NHP	1,199	4.46	9.89	34.12

^*^PI, Percentage inhibition.

**Table 2:** Henipavirus–positive sera detected by indirect ELISA and cELISA.

**Animals**	**Inoculated viruses**	**Indirect ELISA (Pos./Total)**	**cELISA (Pos./Total)**
Pigs	NiV–B	19/19	19/19
	NiV–M	25/25	25/25
Non-human primates	NiV–B	8/8	8/8
	NiV–M	9/9	9/9
	HeV	8/8	**7/8**
Miniature Pigs	HeV	2/2	2/2

Positive sera from Nipah virus (NiV) and Hendra virus (HeV) experimentally inoculated pigs, minipigs and NHPs were collected from 14 dpi to 35 dpi. The sera were tested using NiV–G indirect ELISA and cELISA.

**Figure 4:**
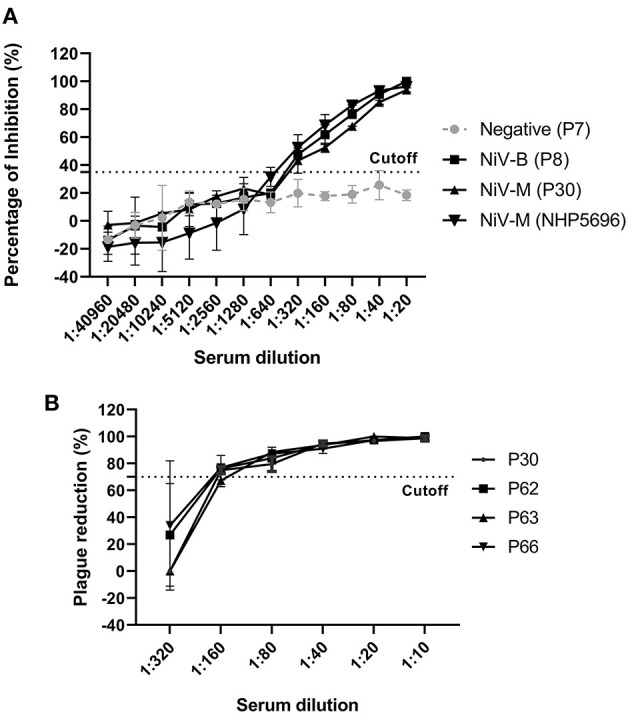
Analytic sensitivity of NiV cELISA and Plaque Reduction Neutralization Test (PRNT) **(A)** NiV (Bangladesh, Malasia) positive sera (28 dpi) from experimentally inoculated pigs and NHPs were 2-fold diluted and tested using the NiV cELISA, the values are the mean of percentage inhibition in duplicates with error bars, and **(B)** NiV–M–positive sera were 2-fold diluted and tested using PRNT. A PRNT result of ≥ 70% plaque reduction was considered a positive result. The values are the mean of replicates with error bars.

To determine the analytical sensitivity, NiV– and HeV–positive sera (28 dpi) were serially diluted 2–fold and tested with our NiV cELISA. NiV–M and –B sera showed similar percent inhibition curves and positive results at a serum dilution of 1:320 (Figure 4A). The results showed that the NiV cELISA was equally sensitive for the detection of both NiV strains. Four NiV–M positive sera were also tested using PRNT to compare the analytical sensitivity with cELISA. Three of the four pigs showed positive results at a serum dilution of 1:160, while one serum was positive at a dilution of 1:80 (Figure 4B). Therefore, the analytical sensitivity of cELISA for NiV–M antibody is one dilution higher than that of PRNT. Here only NiV–M positive sera were tested using PRNT, samples from NiV–B and HeV may need to be analyzed using PRNT.

### Antibody response kinetics using the NiV cELISA and plaque reduction neutralization assay

**Figure 5:**
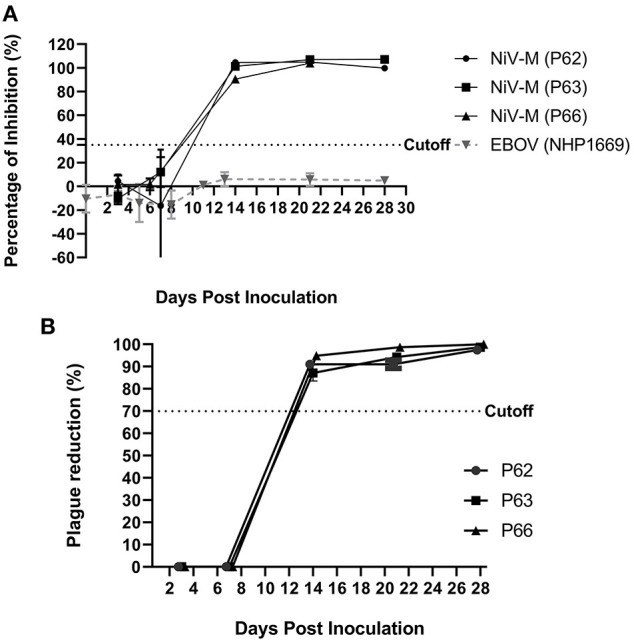
Antibody response kinetics to NiV–G using the NiV cELISA and plaque reduction neutralization assay (PRNT). Serum samples from pigs (#62, 63, 66) and NHPs experimentally infected with NiV–M or Ebola virus (EBOV) were collected at 4–7 day intervals from 3 to 28 days post inoculation (dpi) and tested using **(A)** NiV cELISA. Values higher than 35% inhibition were considered as positive results **(B)** PRNT results for NiV sera only. A PRNT result of ≥70% plaque reduction was considered a positive result. The values are the mean of duplicates with error bars.

To determine the kinetics of NiV seroconversion, serum samples from NiV–M–inoculated pigs (#62, 63, and 66) were collected at 3–7 day intervals from 3 to 28 dpi and tested using the cELISA and PRNT. Using the NiV cELISA, NiV antibodies were undetectable until 8 dpi. All three pigs demonstrated positive antibody responses at 14 dpi and remained positive until the end of the experiment (28 dpi) (Figure 5A). Due to a lack of serum samples from 9 dpi to 13 dpi, the exact time when seroconversion occurred is unknown. To compare the cELISA results with the gold–standard serological test, samples were analyzed using PRNT. Similar to cELISA, all samples were negative by 8 dpi and seroconverted to positive after 14 dpi using PRNT (Figure 5B). Cohen's kappa coefficient was equal to 1, which indicates a complete agreement between our NiV cELISA and PRNT. The kinetics of serological responses to NiV–B and HeV were not tested due to the lack of serum samples. None of the Ebola virus–positive sera (0 to 28 dpi) showed a positive antibody response in the NiV cELISA, indicating that the NiV cELISA is specific for henipavirus antibody detection.

## Discussion

The development of reliable diagnostics is key to the prevention and management of zoonotic disease outbreaks. Serological testing is used to confirm viral infection and for epidemiological surveillance. Here, we report the development and evaluation of a cELISA for the detection of antibodies to NiV and HeV in different animal species. To develop the cELISA, recombinant NiV–G was used as the antigen. The advantage of using recombinant proteins as antigens rather than viral particles is that they do not require preparation in BSL−4 laboratories. Furthermore, one report found that the recombinant NiV nucleocapsid protein (N)–based IgM ELISA was more sensitive than the inactivated virus–based ELISA (13). Previous reports have shown that recombinant NiV–G exhibited epitopes and structure necessary for specific antigen–antibody recognition (22). Since NiV and HeV glycoproteins are highly conserved and share 80% amino acid identity (23), and positive sera from NiV and HeV inoculated animals cross–reacts with NiV–G (22), so recombinant NiV–G is an ideal antigen for immunoassay. As expected the NiV– and HeV–positive sera showed reactivity to the recombinant NiV–G. The results confirmed that the recombinant NiV–G contained the conserved immunodominant epitope of henipaviruses and could be used as an antigen for the development of our NiV cELISA.

The use of mAbs in serodiagnosis provides a stable supply of reagents. Unlike polyclonal antisera, once the mAb has been produced, there is no need to repeat the production using virus immunization in animals. A NiV–specific mAb F20NiV−65 was previously generated and characterized. This mAb reacted with the recombinant NiV–G, but did not cross–react with the Ebola virus glycoprotein, indicating that this mAb is NiV–G specific (16). When tested in our cELISA, this mAb showed a strong inhibitory effect on the binding of NiV and HeV polyclonal antibodies to NiV–G. Therefore, the mAb F20NiV−65 was chosen as the competitor in the cELISA for the detection of NiV and HeV antibodies. Fischer et al. reported (14) that NiV–positive sera from pigs were more reactive to recombinant NiV–G than from other animal species in a NiV–G indirect ELISA. This means that more porcine NiV antibodies were detected using recombinant NiV–G antigen than other animal species. However, in the current cELISA, no significant differences were observed in samples from pigs, minipigs and NHPs. Although the recombinant NiV–G used in the cELISA was derived from NiV–B, it was cross–recognized by all NiV (M, B) and HeV positive sera. The results show that this cELISA can be used to detect NiV and HeV antibodies, rather than using recombinant NiV–G or HeV–G to detect NiV and HeV homologous antibodies, respectively, as in an indirect ELISA (14).

The diagnostic sensitivity and specificity of this NiV cELISA was 98.59 and 99.92%, respectively. Diagnostic specificity of the cELISA is comparable to previously published reports of blocking ELISA for NiV antibody detection (24), but higher than the indirect ELISA (95.8–99%) (1, 13, 25, 26). A similar report indicated that a cELISA was superior to an indirect ELISA in the detection of anti–Bluetongue Virus antibody in sera and whole blood samples from both cattle and sheep early after infection with Bluetongue Virus (27). Although our NiV cELISA has high diagnostic sensitivity, one of the 71 true positive serum samples was identified as a negative result. One possible explanation for the false–negative results could be due to the difference in epitope recognition between indirect ELISA and cELISA. The mAb used in the cELISA recognizes a single epitope, but the indirect ELISA can detect multiple epitopes present on the NiV–G protein.

One disadvantage of indirect ELISA is the need to change the secondary antibody depending on the animal species or human. Since both NiV and HeV are zoonotic viruses, these viruses can infect different animal species and humans. Any reagent change requires re–titration and verification, which is inconvenient. Whereas, cELISA overcomes this problem and does not require changing reagents to test a variety of samples from animals and possibly humans. But this needs to be further confirmed by testing human serum samples. The analytical sensitivity of cELISA for NiV antibody is one dilution higher than that of PRNT. However, the PRNT assay detects neutralizing antibodies, whereas the cELISA developed in this assay detects NiV–G binding antibodies. Thus, the analytical sensitivity of cELISA is higher than that of PRNT. Therefore, PRNT may not be the best method to compare the assay sensitivity of new cELISAs.

Determination of the immune response kinetics after NiV and HeV infection would help in understanding the course of infection at different stages of the disease. Using the cELISA, serum samples from three NiV–M–inoculated pigs demonstrated positive antibody responses at 14 dpi and remained positive until the end of the experiment (28 dpi). Our cELISA was specific for NiV antibodies as it showed negative results for an Ebola virus–positive serum sample. Similar to cELISA, samples of three pigs were seroconverted to positive after 14 dpi using PRNT. Our results are consistent with the report published by Hanna et al. (28) which indicated a positive result using PRNT 14 days after virus inoculation. Generally, cELISA can detect all antibody isotypes (IgM and IgG) as long as antibodies against the NiV–G protein are present in samples. It was reported symptoms usually appear 4–14 days after exposure to NiV (29). Anti–NiV IgM peaks were detected in serum after 9 days of illness and IgG peaks after 17 days of illness based on hospital admittance (9). Therefore, the 14–day seroconversion of the experimentally inoculated animals detected by our cELISA was similar to the time of IgM peak. IgM ELISA is commonly used as a first–line NiV serological diagnostic test (9).

For diagnostic test development, full validation is important before being used for diagnosis. However, validation of zoonotic testing has been limited by the lack of positive samples. In this study, we used henipavirus–positive sera (NiV, HeV) from pigs, and minipigs, but not human samples. It is possible that using animal samples may have a risk that the immune response to NiV infections may differ from those found in humans (9). Therefore, we included serum samples from NHPs, and the results showed that our NiV cELISA also detected NiV and HeV antibodies in NHP sera, indicating that the cELISA can be used on samples from different animal species. Although, we did not have the opportunity to detect NiV antibodies from human samples, it has been reported that cELISA can rapidly detect Trichinella–specific antibodies in sera from two different host species (human and pig) (30). Due to the lack of field sera and only a small amount of positive laboratory sera available, additional samples will be tested using cELISA if available in the future for a full validation of this cELISA.

## Conclusion

A cELISA for NiV and HeV serodiagnosis was developed using the recombinant NiV–G and a NiV–G specific mAb. This cELISA could be used as a screening test for suspect NiV and HeV cases and for epidemiological surveillance of henipavirus infections in different animal species and humans, although further validation is required. The main improvements of our NiV cELISA over previously published similar assays are (1) the use of recombinant NiV–G, which can be performed in the CL2 laboratory without viral antigen preparation; (2) the detection of antibodies against both NiV and HeV, and (3) the ability to detect NiV and HeV antibodies in different animal species without changing reagents under the same conditions. Since the cELISA identifies specific antibodies to the G protein, which is a protective antigen used in different vaccines, the assay can be used for post–vaccination monitoring to determine seroconversion.

## Data availability statement

The raw data supporting the conclusions of this article will be made available by the authors, without undue reservation.

## Ethics statement

The animal study was reviewed and approved by the Animal Care Committee of the Canadian Science Centre for Human and Animal Health.

## Author contributions

Conceptualization: MY and LB. Methodology: WZ, GS, MP, and TT. Validation: WZ, GS, and MP. Formal analysis: MY, WZ, and GS. Investigation and writing – original draft preparation: MY and WZ. Resources and funding acquisition: MY, LB, BP, DK, and SB. Data curation: MY, WZ, GS, and MP. Writing – review and editing: MY, LB, DK, SB, WZ, GS, BP, and TT. Visualization: MY, WZ, and LB. Supervision: MY, LB, BP, and DK. Project administration: MY. All authors contributed to the article and approved the submitted version.
